# Direct Sinus Floor Elevation With Simultaneous Implant Placement for Rehabilitation of the Atrophic Maxilla: A Case Report

**DOI:** 10.7759/cureus.117527

**Published:** 2026-10-06

**Authors:** Uttam Shetty, Rohit Pokalwar, Swapnali Mhatre, Reema Srichand, Mridula Joshi

**Affiliations:** 1 Department of Prosthodontics, Bharati Vidyapeeth (Deemed to Be University) Dental College and Hospital, Navi Mumbai, IND; 2 Department of Prosthodontics, Crown and Bridge, and Implantology, Bharati Vidyapeeth (Deemed to Be University) Dental College and Hospital, Navi Mumbai, IND

**Keywords:** atrophic posterior maxilla, dental implants, direct sinus lift, lateral-window sinus augmentation, sinus floor elevation

## Abstract

Rehabilitating the posterior maxillary region with dental implants can be challenging because of inadequate residual bone height from alveolar ridge resorption and progressive pneumatization of the maxillary sinus. When residual vertical bone height is inadequate for conventional implant placement, maxillary sinus floor elevation can provide the additional vertical bone required for implant-supported rehabilitation. The lateral-window approach permits direct visualization and controlled elevation of the Schneiderian membrane and is particularly useful when substantial vertical augmentation is required.

A patient reported to the Department of Prosthodontics, Crown and Bridge, and Implantology with multiple missing teeth and retained root stumps in the maxillary arch. Clinical examination revealed missing teeth, retained root stumps, and root-canal-treated, grossly carious teeth requiring definitive restorations. Cone-beam computed tomography examination demonstrated approximately 4 mm of residual vertical bone height and 6 mm of buccolingual ridge width in the right posterior maxilla. A staged treatment plan was formulated. A direct sinus lift was performed using a lateral-window approach, followed by simultaneous placement of implants. Both implants achieved good primary stability at the time of placement. Definitive zirconia restorations were subsequently provided for the root-canal-treated teeth and implant-supported sites.

This case demonstrates a staged approach for complex posterior maxillary rehabilitation using direct lateral-window sinus augmentation, simultaneous implant placement, controlled graft management, and definitive zirconia prostheses. Within the limitations of the short follow-up period, satisfactory clinical and functional rehabilitation was achieved.

## Introduction

Implant rehabilitation of the maxillary posterior region presents a clinical challenge due to progressive alveolar ridge resorption and sinus pneumatization following tooth loss. The resultant reduction in residual alveolar bone height may compromise the placement of implants of clinically appropriate and in an optimal prosthetically driven position, thereby necessitating augmentation of the deficient posterior maxillary site.

Elevation of the maxillary sinus floor is a predictable and widely accepted surgical approach to increase the available vertical bone dimension and facilitate implant placement in the atrophic posterior maxilla. Since the initial description of sinus augmentation by Boyne and James, several modifications have been introduced concerning surgical access, membrane management, graft materials, and implant-placement protocols [1].

Sinus augmentation can generally be performed using either a transcrestal or lateral-window approach. Technique selection depends on factors such as residual bone height, ridge anatomy, the number and position of implants, sinus morphology, and the amount of augmentation required. The lateral approach allows direct visualization and controlled elevation of the Schneiderian membrane and is commonly indicated when substantial bone-height augmentation is required.

Pjetursson et al. reported favorable implant outcomes following sinus floor elevation, supporting the predictability of sinus augmentation for Implant-supported reconstruction of the atrophic posterior maxilla, with a three-year implant survival of 90.1% [2]. Long-term evidence has further supported the survival of implants placed in augmented sinuses. At greater than or equal to three years of functional loading, survival rates were 93.7% and 97.2% for implants placed using the lateral window and transalveolar approaches, respectively [3].

Graft material selection is another important consideration. Deproteinized bovine bone mineral (DBBM) has been extensively evaluated in sinus augmentation procedures and has demonstrated favorable clinical outcomes. Jensen et al. demonstrated favorable implant survival rates following sinus augmentation using Bio-Oss® (Geistlich Pharma AG, Switzerland), either alone or combined with autogenous bone [4]. Barrier membranes have been incorporated into lateral-window sinus augmentation procedures, with studies reporting improved graft containment and a conducive environment for new bone formation [5].

This case report presents the staged rehabilitation of maxillary distal extension bases in a patient with multiple missing teeth and endodontically treated teeth. The report describes management of an atrophic posterior maxilla using a direct lateral-window sinus elevation procedure in conjunction with immediate implant placement.

## Case presentation

A 59-year-old male patient reported to the Department of Prosthodontics with a chief complaint of missing teeth in the upper arch and difficulty in mastication. Clinical examination revealed missing teeth in relation to 15, 16, and 24. Retained root stumps were present in relation to 17 and 25. Teeth 13, 14, 23, and 26 were previously root-canal treated and required definitive full-coverage restorations (Figure 1).

**Figure 1:**
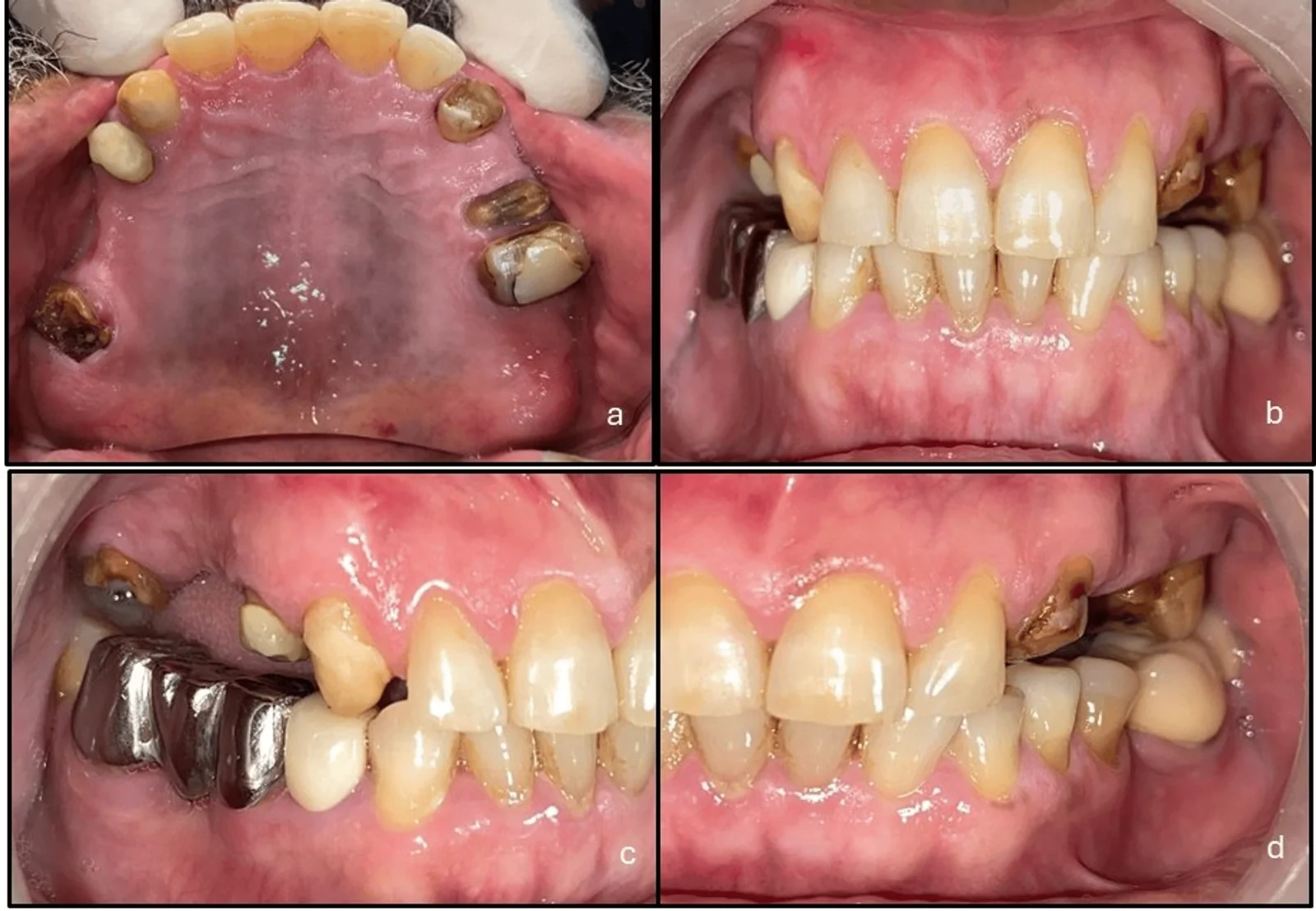
Preoperative intraoral photographs demonstrating the partially edentulous maxillary arch with retained root stumps: (a) maxillary occlusal view, (b) frontal view, (c) right lateral view, and (d) left lateral view

Clinical evaluation of the edentulous regions included assessment of ridge morphology, soft-tissue condition, available prosthetic space, and occlusal relationships. A preoperative cone-beam computed tomography examination was performed for three-dimensional evaluation of the maxillary alveolar ridges and maxillary sinus.

The residual vertical bone height beneath the right maxillary sinus was approximately 4.37 mm, while the buccolingual ridge width was approximately 6.88 mm (Figure 2). The available vertical bone height was considered inadequate for placement of the planned implants without sinus augmentation. The sinus membrane appeared normal in thickness, with no evidence of polyps or other pathological growths. A limiting septum was present distally in the region of tooth 17, which was advantageous in providing a distal limit for the grafting material. Therefore, a direct sinus lift using a lateral-window approach was selected.

**Figure 2:**
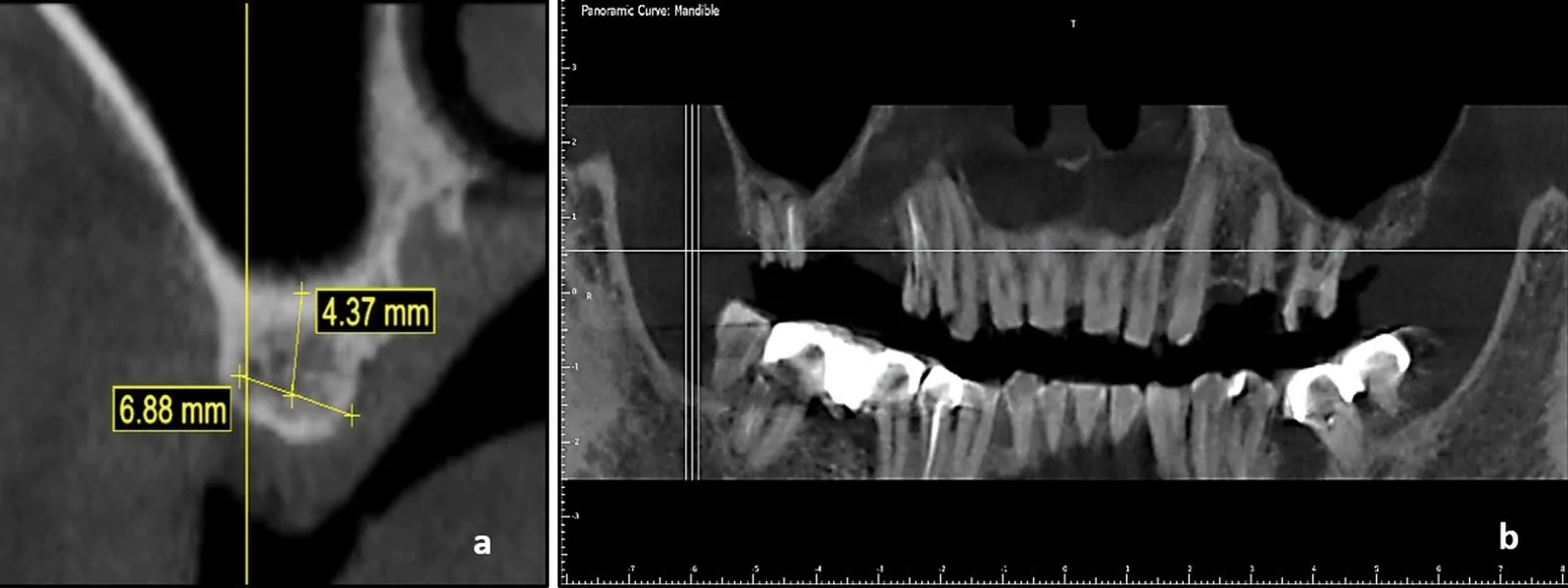
Preoperative CBCT residual vertical bone height and buccolingual ridge width in the right posterior maxilla. (a) Cross-sectional view. (b) Panoramic view CBCT: cone-beam computed tomography

A multistage treatment was planned and implemented. The treatment objectives were removal of root stumps, implant-supported rehabilitation of the edentulous sites, augmentation of the deficient posterior maxillary region, definitive restoration of the root-canal-treated teeth, and establishment of satisfactory function, occlusion, and esthetics. In the left posterior maxillary region, a conventional immediate implant was planned without sinus lift because of adequate bone availability, followed by delayed loading. In the right posterior maxillary region, rehabilitation was planned with a direct sinus lift and immediate implant placement, followed by delayed loading. The treatment plan and possible complications were explained, and informed consent was obtained from the patient.

The first surgical procedure was performed in the posterior maxillary region involving the edentulous sites 24 and 25. Following administration of local anesthesia, the retained root stump at 25 was removed. Implant osteotomies were prepared according to the planned implant positions, and two Osstem implants measuring 4 × 10 mm were placed (Figure 3). Immediate implant placement was carried out in the left posterior maxilla without sinus lift, given the presence of adequate residual bone. Both implants achieved good primary stability with 45 N·cm of insertion torque. The surgical sites were irrigated; the soft tissues were repositioned and sutured to obtain satisfactory primary closure.

**Figure 3:**
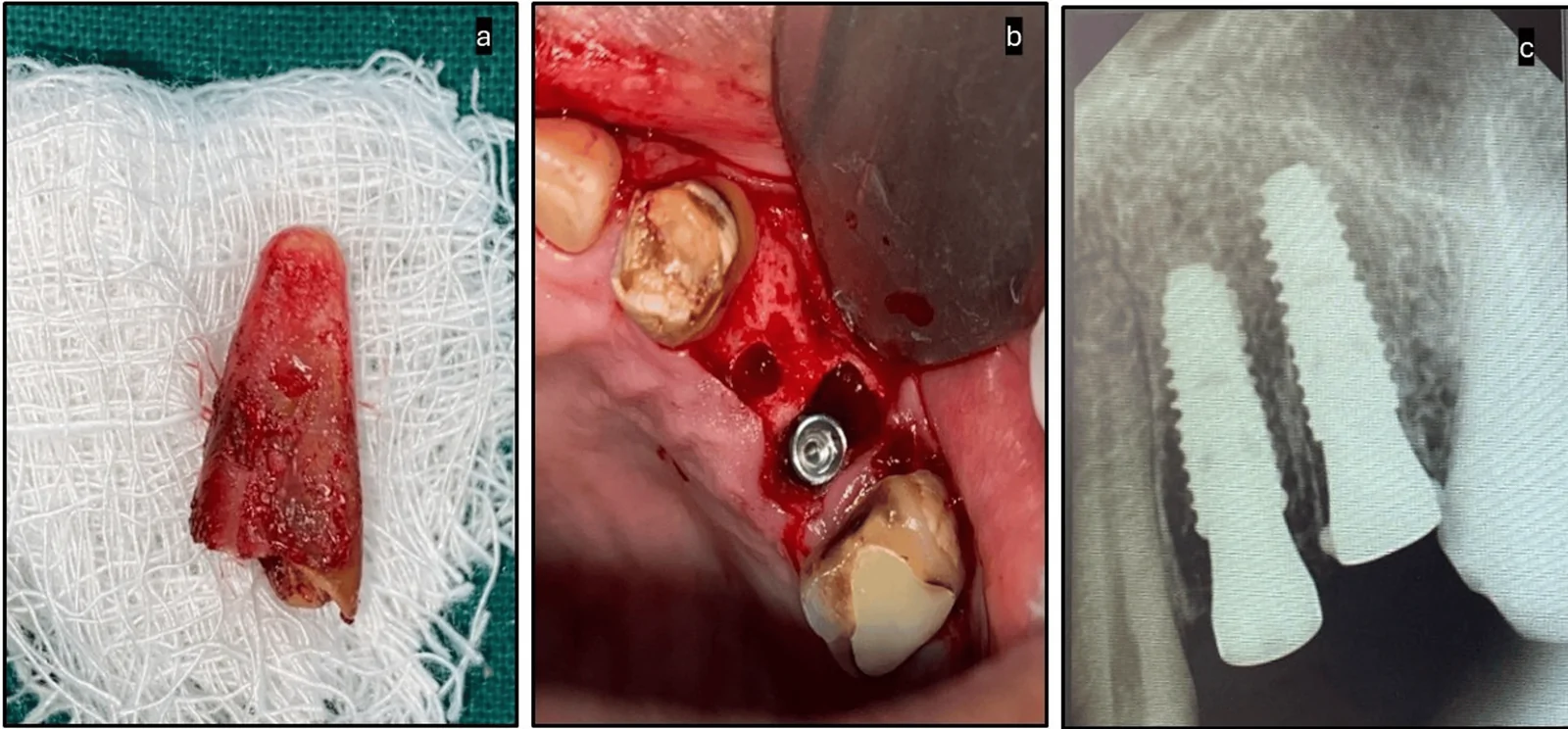
Surgical procedure: (a) extraction of tooth 25, (b) immediate implant placement at sites 24 and 25, and (c) postoperative radiograph

The second surgical procedure was performed in the right posterior maxillary region involving sites 15 and 16. Following administration of local anesthesia, the lateral wall of the maxilla was exposed by reflecting a mucoperiosteal flap using the Modified Lateral Osteotome technique with the (LAS)KIT® (OSSTEM IMPLANT Co., Ltd., Haeundae-gu, Busan, Republic of Korea). The LAS Kit consists of dome, core, and side-wall drills, specifically designed to prepare and create the lateral window during maxillary sinus floor elevation. These drills can be used safely at a recommended speed of 1200-1500 rpm with a stopper system to control drilling depth. Compared with the conventional Caldwell-Luc approach, this technique allows creation of a smaller, more conservative lateral window, minimizing unnecessary exposure of the Schneiderian membrane and reducing surgical trauma.

We prepared a window using the LAS Kit in the lateral bony wall of the maxillary sinus to access the sinus cavity. Using appropriate sinus membrane elevators, the Schneiderian membrane was atraumatically lifted from the sinus floor. The membrane was elevated to create an adequate subantral space for graft and implant placement (Figure 4).

**Figure 4:**
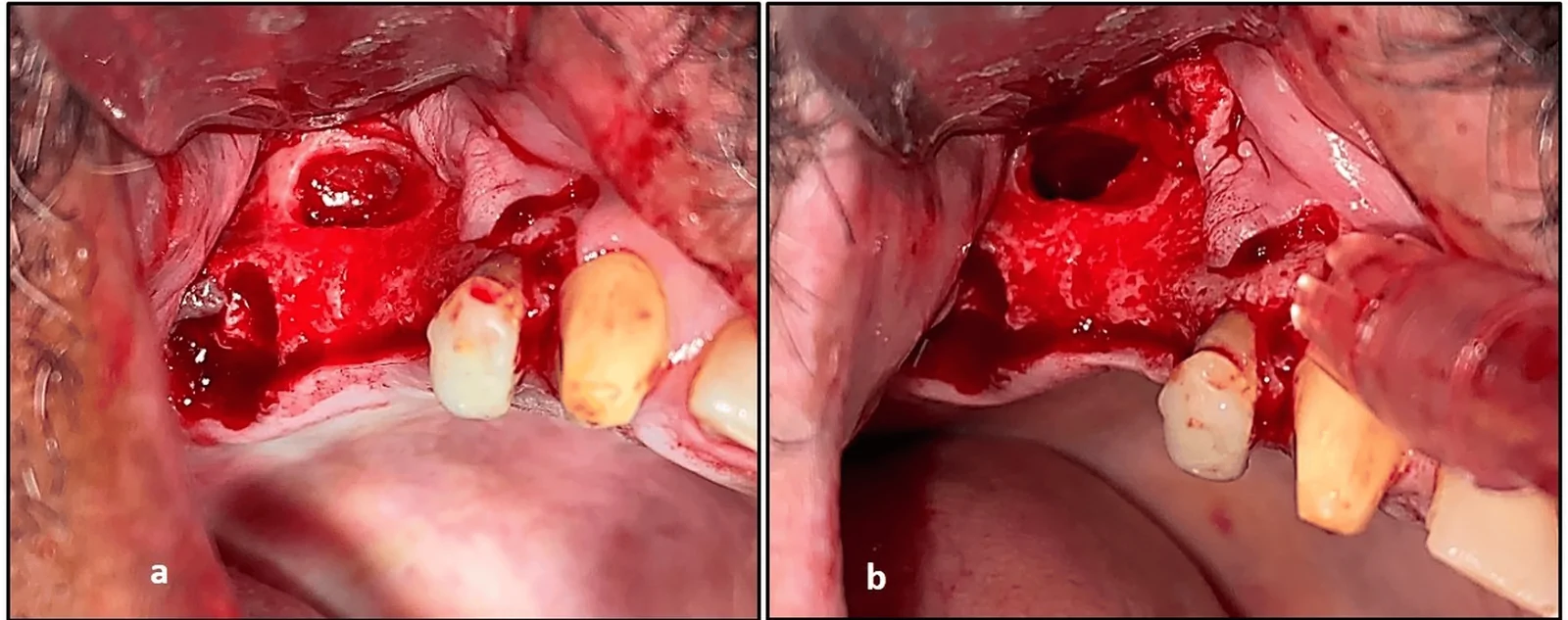
Exposure of the maxillary sinus. (a) Lateral window technique. (b) Membrane elevation

The membrane was carefully elevated during the procedure. Membrane perforation is one of the principal complications associated with lateral sinus window preparation [6-8]. Hence, a resorbable membrane was first positioned within the surgical site (Figure 5). Approximately 1.5 cc of bone graft material Geistlich Bio-Oss® was placed into the prepared subantral compartment.

**Figure 5:**
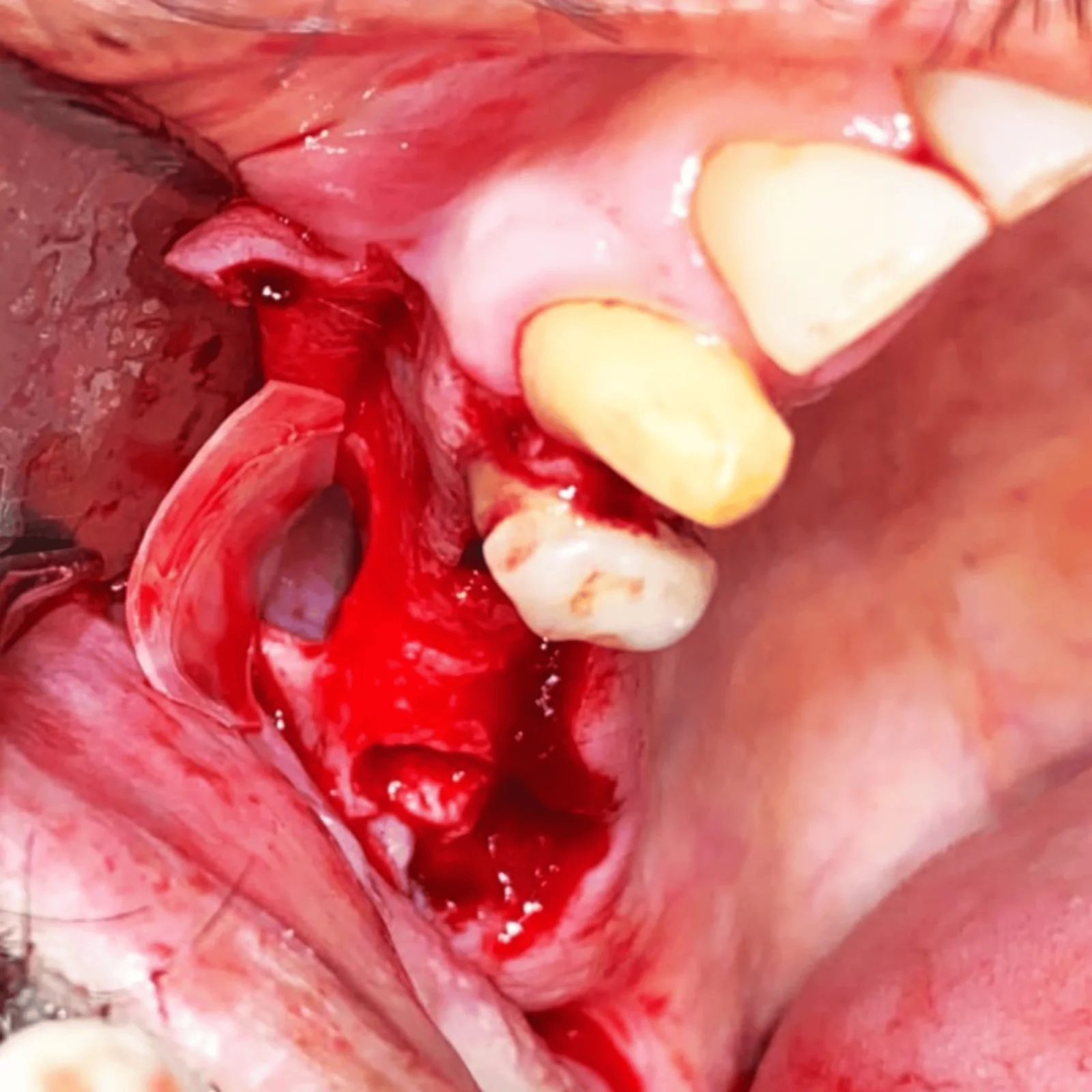
Membrane placement

The graft material was gently packed within the sinus space while avoiding excessive pressure against the elevated Schneiderian membrane. The use of DBBM in sinus augmentation has been extensively studied, with favorable implant survival reported when Bio-Oss® is used alone or in combination with autogenous bone [4].

Following graft placement, the retained root stump at 17 was carefully removed using an atraumatic extraction technique. Care was taken to minimize unnecessary trauma to the surrounding tissues and to avoid compromising the augmented sinus region.

Following extraction of 17, implant osteotomies were prepared at the planned positions of 15 and 16. Osstem implants measuring 4 × 10 mm at site 15 and 4.5 × 8.5 mm at site 16 were placed. Both implants achieved good primary stability with an insertion torque of 45 N·cm (Figure 6).

**Figure 6:**
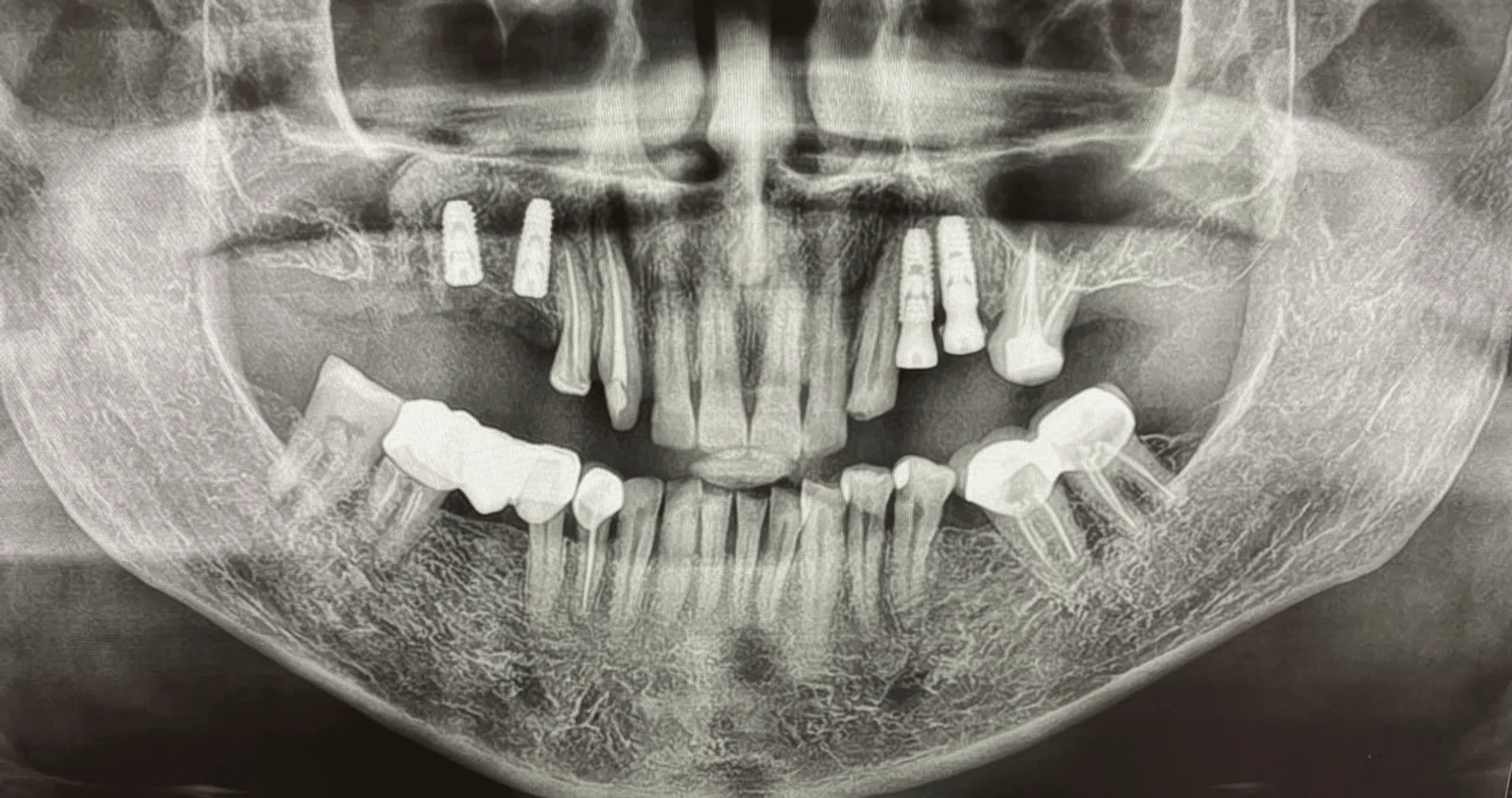
Simultaneous placement of Osstem implants at sites 15 and 16 following direct sinus augmentation

The implants were positioned according to the available anatomy and prosthetically planned positions. The surgical site was sutured after repositioning of the flap. The patient was initially followed at eight days to assess soft-tissue healing and the surgical sites. Subsequent clinical evaluations were performed at three, four, and five months. The implant sites and augmented region were evaluated for clinical healing, implant stability, soft-tissue health, and postoperative or sinus-related complications.

One month after the second surgical procedure, definitive zirconia crowns were placed on the previously root-canal-treated teeth. The crowns were evaluated for marginal adaptation, proximal contacts, occlusal relationship, contour, and esthetic integration.

At five months following implant placement, the second-stage implant procedure was initiated. All four implants demonstrated satisfactory clinical stability. The implants were evaluated clinically and radiographically for stability, and implants with high implant stability quotient values (>70) were considered suitable for definitive prosthetic rehabilitation. Definitive zirconia implant-supported crowns were fabricated and delivered at these sites six months postoperatively (Figures 7, 8). The restorations were evaluated for marginal adaptation, proximal contacts, occlusal relationships, emergence profile, interproximal contours, patient comfort, and functional integration. Occlusal adjustments were performed where necessary.

**Figure 7:**
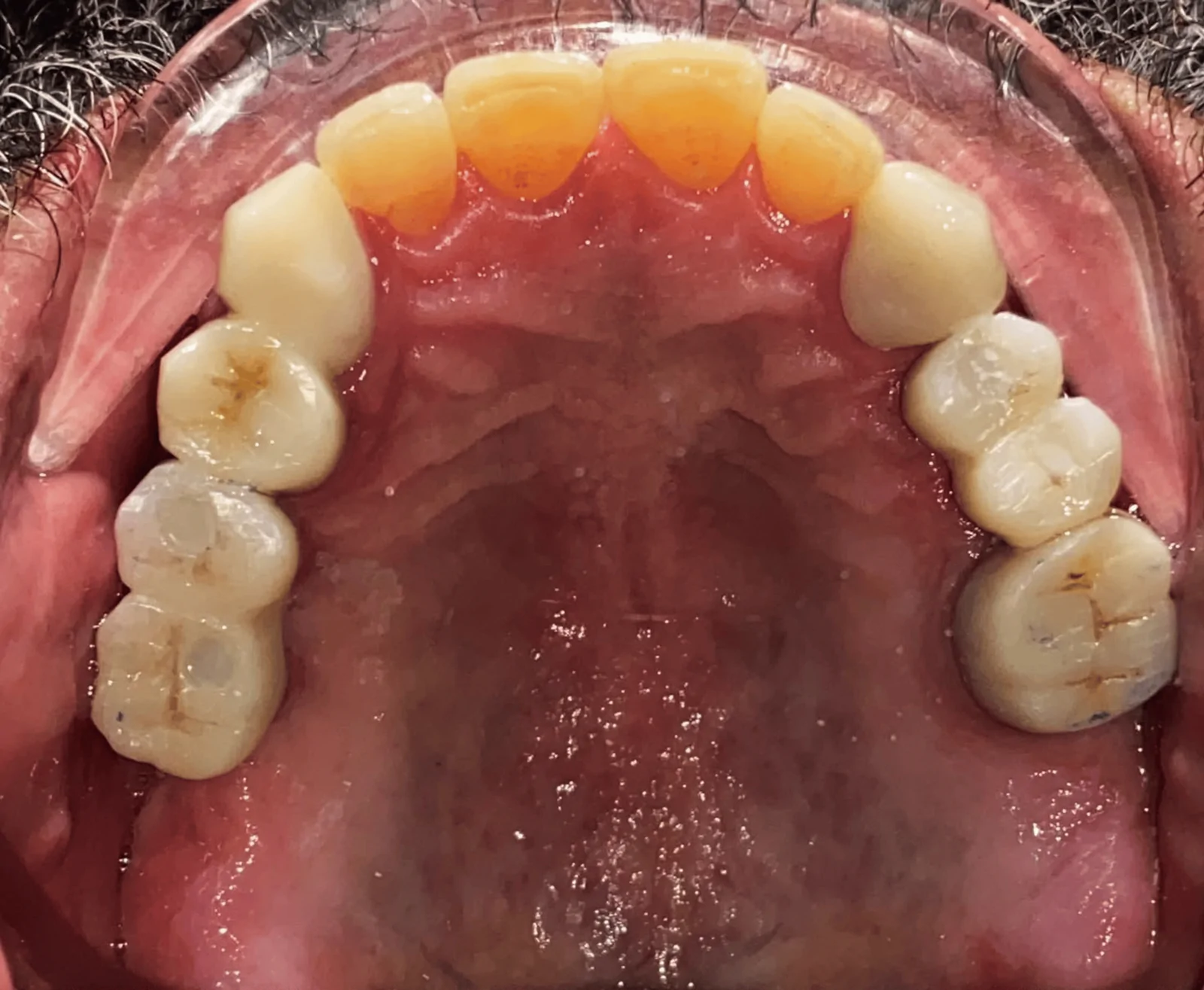
Definitive zirconia implant-supported crowns at maxillary posterior region

**Figure 8:**
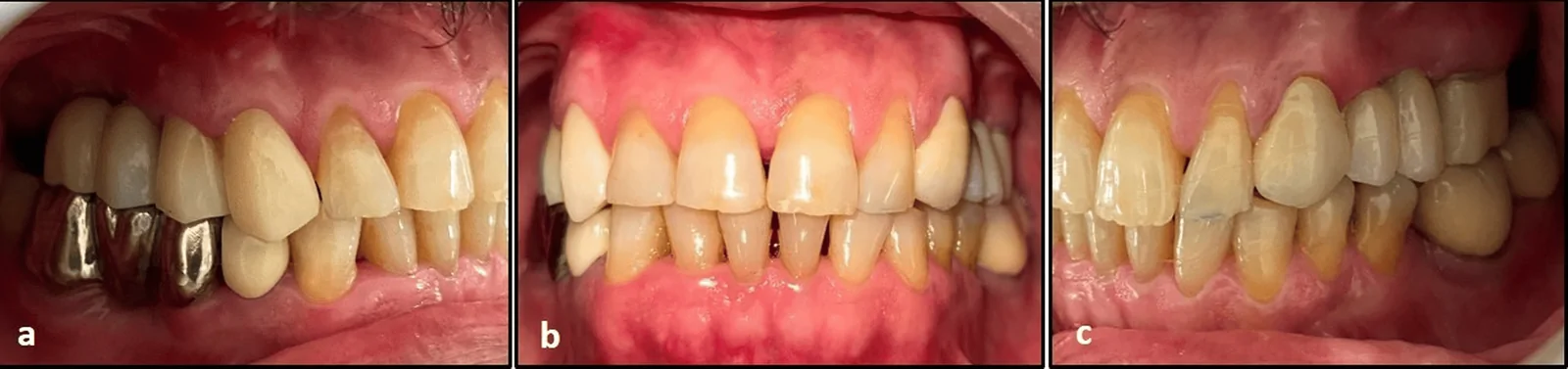
Final intraoral view following comprehensive bilateral posterior maxillary rehabilitation. (a) Right lateral view. (b) Frontal view. (c) Left lateral view

Following the delivery of the definitive implant-supported zirconia crowns, the patient was recalled at one day, eight days, and one month for initial follow-up. During these visits, patient comfort, occlusal contacts, proximal contacts, soft-tissue response, crown stability, oral hygiene, and functional adaptation were evaluated. Further follow-up visits were conducted at three months and one year. The patient demonstrated satisfactory clinical adaptation to the definitive prosthetic rehabilitation throughout follow-up.

## Discussion

The present case involved rehabilitation of a compromised posterior maxillary region with missing teeth, retained root stumps, and inadequate vertical bone availability. The main challenge was the reduced residual bone height in the first maxillary molar region, which was approximately 4 mm. This amount of available bone was considered insufficient for predictable placement of the planned implants. Therefore, maxillary sinus floor elevation was considered necessary to increase the available vertical bone dimension and permit placement of implants in a prosthetically favorable position.

A direct sinus lift was selected due to the need for a significant quantity of vertical bone augmentation of more than 5 mm to facilitate implant placement at two adjacent sites. A transcrestal approach, or indirect sinus lift, is less invasive and suitable when only limited sinus elevation is required due to anatomic limitations; attempting to elevate the sinus membrane beyond a small range (more than 5 mm) dramatically increases the risk of tearing or perforating the membrane [7]. The direct approach provides greater access and allows controlled elevation of the Schneiderian membrane under direct visualization [8].

Pjetursson et al. reported favorable implant outcomes following sinus floor elevation, supporting the use of sinus augmentation for implant rehabilitation of the atrophic posterior maxilla [2]. In the present case, this was particularly advantageous because adequate vertical space had to be created for simultaneous implant placement at both 15 and 16. The direct approach by the lateral-window technique therefore provided better surgical control over membrane elevation and graft placement in this region. It was more appropriate than a transcrestal approach because of the amount of augmentation required for multiple adjacent implant sites [7,9].

Following elevation of the Schneiderian membrane, bone grafting was performed to occupy and stabilize the newly created subantral compartment and to provide an osteoconductive scaffold for bone formation. Autogenous graft is the material of choice for any augmentation, but for sinus lift we can also use allograft, xenograft, or alloplast bone graft. Maintaining the space created beneath the elevated membrane is important for predictable regeneration of the augmented region. Approximately 1.5 cc of Geistlich Bio-Oss® bone graft material was placed in the augmented region and gently adapted without exerting excessive pressure on the Schneiderian membrane. DBBM graft material has been widely used for sinus augmentation because of its osteoconductive properties and favorable handling characteristics [4,10]. Jensen et al. reported favorable implant survival following sinus augmentation using Bio-Oss® alone or in combination with autogenous bone [4].

A resorbable membrane was placed before introduction of the graft material. The membrane was used to provide a barrier and assist in maintaining the grafted compartment while supporting a controlled environment for bone regeneration. Membrane coverage has been investigated in lateral-window sinus augmentation, and evidence suggests that barrier membrane application may provide favorable conditions for bone regeneration and graft containment [5,11]. In the present case, placement of the membrane before grafting was therefore intended to establish a controlled grafting compartment and minimize displacement of the particulate graft material.

Simultaneous implant placement was selected because adequate primary stability could be achieved at the time of surgery. The implants placed at 15 and 16 achieved an insertion torque of 45 N·cm and demonstrated good primary stability. When adequate primary stability can be obtained, simultaneous implant placement can reduce overall treatment duration and eliminate the need for a separate implant-placement procedure after completing sinus augmentation [9]. It also permits the implants to undergo healing together with the augmented site. In the present case, the residual bone available beneath the sinus, together with the selected implant dimensions, allowed satisfactory primary stability to be achieved; therefore, delayed implant placement was not considered necessary.

The retained root stump at 17 was removed after graft placement using an atraumatic extraction technique. The sequence was selected to minimize unnecessary surgical trauma and to avoid disturbing the newly augmented sinus region. Because the extraction site was adjacent to the grafted area, excessive manipulation could potentially compromise the surrounding tissues or disturb the graft material. A careful atraumatic technique was therefore used to minimize tissue trauma and preserve the surgical environment.

A staged treatment approach was adopted because the patient required rehabilitation of multiple sites with different surgical and prosthetic requirements. The first surgical procedure involved extraction of the retained root followed by immediate implant placement at the left maxillary posterior region, whereas the second procedure addressed the sinus-deficient right maxillary posterior region. This approach allowed the procedures to be performed in a controlled manner and facilitated monitoring of healing and implant stability. It also allowed the root-canal-treated natural teeth to be restored independently while the implants underwent the planned healing period.

The use of zirconia for the definitive restorations provided a metal-free restorative option and allowed a consistent restorative material to be used for both the natural teeth and implant-supported restorations. Zirconia crowns were placed on the root-canal-treated teeth approximately one month after the second surgical procedure, while the implant-supported zirconia crowns were delivered after five months of healing. This sequence allowed sufficient time to evaluate implant stability and healing of the augmented region before definitive implant prosthetic rehabilitation.

The implants placed at 15, 16, 24, and 25 achieved good primary stability with an insertion torque of 45 N·cm. The patient was followed at 10 days, three months, four months, and five months following the surgical procedures, and no sinus-related complication was reported during the documented follow-up. After definitive prosthetic rehabilitation, the patient was reviewed at 1, 8, and 15 days, demonstrating satisfactory clinical adaptation and functional rehabilitation.

The present case has limitations that should be considered when interpreting the outcome. It represents a single clinical case; therefore, the findings cannot be generalized to all patients undergoing sinus augmentation. The postprosthetic follow-up period was limited, preventing assessment of long-term implant survival, peri-implant marginal bone stability, sinus health, graft maturation, and prosthetic longevity. No histological evaluation was performed; therefore, the quality and maturation of the newly formed bone within the augmented sinus could not be directly assessed.

Overall, this case demonstrates that treatment selection in the severely resorbed posterior maxilla should be based on residual bone height, the amount of vertical augmentation required, sinus anatomy, the number and position of implants, and the ability to achieve primary implant stability. In this patient, the combination of a direct lateral-window sinus lift, careful Schneiderian membrane elevation, membrane placement, bone grafting, atraumatic extraction, and simultaneous implant placement provided a clinically satisfactory approach for posterior maxillary rehabilitation within the limitations of the reported follow-up period [2-5].

## Conclusions

This case report demonstrates the successful rehabilitation of an atrophic posterior maxilla using a staged surgical and prosthetic approach incorporating direct lateral-window maxillary sinus floor elevation with simultaneous implant placement. Despite the limited residual vertical bone height, appropriate sinus augmentation and controlled graft placement provided adequate conditions for implant rehabilitation and subsequent definitive prosthetic restoration. The satisfactory clinical and functional outcome emphasizes the importance of comprehensive three-dimensional diagnosis, prosthetically driven treatment planning, careful selection of the sinus augmentation technique, meticulous management of the Schneiderian membrane, controlled graft handling, achievement of adequate primary implant stability, and atraumatic surgical techniques.

The case further highlights the importance of coordinated sequencing between the surgical and prosthetic phases in the management of complex posterior maxillary rehabilitation. Appropriate healing and osseointegration periods followed by definitive prosthetic restoration contributed to a satisfactory functional outcome. Within the limitations of the relatively short follow-up period, no clinically significant complications were observed. Direct lateral-window sinus floor elevation with simultaneous implant placement may therefore be considered a viable treatment approach in appropriately selected patients with posterior maxillary atrophy and inadequate residual bone height. However, meticulous case selection and surgical execution are essential, and long-term clinical and radiographic follow-up is required to evaluate the stability of the augmented sinus, peri-implant hard and soft tissues, implant function, and longevity of the definitive prosthetic rehabilitation.
